# The Moderating Effects of “Dark” Personality Traits and Message Vividness on the Persuasiveness of Terrorist Narrative Propaganda

**DOI:** 10.3389/fpsyg.2022.779836

**Published:** 2022-07-08

**Authors:** Kurt Braddock, Sandy Schumann, Emily Corner, Paul Gill

**Affiliations:** ^1^School of Communication, American University, Washington, DC, United States; ^2^Department of Security and Crime Science, University College London, London, United Kingdom; ^3^Centre for Social Research and Methods, College of Arts and Social Sciences, Australian National University, Canberra, ACT, Australia

**Keywords:** terrorism, radicalization, narratives, narcissism, Machiavellianism, psychopathy, sadism, vividness

## Abstract

Terrorism researchers have long discussed the role of psychology in the radicalization process. This work has included research on the respective roles of individual psychological traits and responses to terrorist propaganda. Unfortunately, much of this work has looked at psychological traits and responses to propaganda individually and has not considered how these factors may interact. This study redresses this gap in the literature. In this experiment (*N* = 268), participants were measured in terms of their narcissism, Machiavellianism, subclinical psychopathy, and everyday sadism—collectively called the Dark Tetrad. Participants were then exposed to a vivid or nonvivid terrorist narrative (or a control message). Results indicate that Machiavellianism interacts with both narrative exposure and narrative vividness to amplify the persuasive effect of terrorist narratives. Neither narcissism, subclinical psychopathy, nor everyday sadism had such an effect. These results highlight the importance of considering the psychological traits of audiences when evaluating proclivity for radicalization *via* persuasion by terrorist narratives.

## Introduction

Many early terrorism researchers argued that an individual’s proclivity for engaging in terrorism is a function of that person’s personality. As the study of terrorism matured, however, experts learned that engagement in terrorist activity cannot be explained by one’s personality traits alone (see Taylor, 1988; Silke, 1998; Borum, 2003; Horgan, 2003, 2008). More recent research has asserted the importance of individuals’ relationships and interactions with those who are already involved with terrorism. Specifically, under the “right” conditions and when exposed to the “right” stimuli (see Bouhana, 2019), certain personality factors can make an individual more prone to violent radicalization. To illustrate, recent research has shown that certain dispositional traits can render an individual more prone to ideological polarization when combined with specific kinds of interactions. For example, Van Prooijen and Krouwel (2019) argued that cognitive simplicity (i.e., black-and-white thinking about their social world), overconfidence in one’s belief system, and intolerance of other belief systems are associated with political extremism. Van Prooijen and Kuijper (2020) demonstrated these relationships experimentally, adding that the feeling that one’s cause is meaningful and willingness to sacrifice oneself or harm others are also associated with extremism. These findings are significant, given that many of these traits are described and emphasized in propaganda perpetuated by extremists that seek to draw others to their cause.

Despite the possibility that the interaction between personality traits and communication facilitates violent radicalization, to date, most research has investigated these risk factors in isolation. For example, past researchers have explored whether individuals who are psychopathic (Cooper, 1976; Hacker, 1976) and/or narcissistic (Lasch, 1979; Post, 1984, 1986; Pearlstein, 1991; Johnson and Feldmann, 1992) were disproportionately likely to join terrorist groups. Additionally, other researchers have demonstrated that exposure to propaganda in support of terrorist organizations can promote support for those organizations (Corman and Schiefelbein, 2008; Halverson et al., 2011; Braddock, 2015; Braddock and Horgan, 2016).

Despite research on the respective effects of personality factors and terrorist messaging, the interaction between them remains unexplored.^1^ This oversight is notable and unfortunate, as the persuasiveness of a message is contingent not only on its content or presentation, but also on the psychological features of those to whom it is presented (e.g., Gerber et al., 2013; Chuang and Tabak, 2015; Lawson et al., 2017; Wall et al., 2019; Zeineddine and Leach, 2021). The present study addresses this gap in the literature.

We explicate a potent form of communication that pervades terrorist propaganda efforts—narratives, and focus on a particular feature of narratives, their vividness. We also examine four personality traits collectively known as the Dark Tetrad (narcissism, Machiavellianism, subclinical psychopathy, and everyday sadism) that may moderate the influence of vivid terrorist narratives on beliefs, attitudes, and behavior in line with terrorist propaganda. With this study, we advance understanding of how, and under what conditions, individuals may be persuaded to support terrorist organizations—and by extension, how these outcomes could be prevented.

### Narrative Persuasion

The study of narrative has produced no shortage of definitions for the term (see Ryan, 2007 for a summary). Though every proposed definition offers some insight into the inherent qualities that narratives possess, we follow Braddock and Dillard (2016, p. 447) to define a narrative as “any cohesive, causally linked series of events that takes place in a dynamic world subject to conflict, transformation, and resolution through non-habitual, purposeful action performed by characters.”

Despite extensive research on narratives, until recently, there had been little evidence to indicate whether they are as persuasive as theorized. Some studies demonstrated that exposure to a narrative would influence an individual’s beliefs and attitudes such that they are in closer alignment with viewpoints espoused therein. Other work found no persuasive effect of narrative, or that narratives induced an *inverse* persuasive effect whereby participants came to adopt viewpoints contrary to the narrative content.

To provide more conclusive evidence as to whether and how narratives persuade, Braddock and Dillard (2016) performed meta-analyses on extant empirical narrative research. Similar to other systematic analyses of narrative forms of persuasion (see Tukachinsky and Tokunaga, 2013; Van Laer et al., 2014; Shen et al., 2015; Oschatz and Marker, 2020) the authors found that across contexts, narrative exposure affects an individual’s beliefs, attitudes, intentions, and behaviors in a manner consistent with the views espoused in the narrative. This finding is relevant in the context of terrorism and political violence, given that terrorist groups’ extensive use of narratives represents a potentially effective form of persuasion.

#### Narrative Vividness

Researchers have long argued that a message’s vividness affects its persuasiveness (see Taylor and Thompson, 1982; Block and Keller, 1997; Escalas, 2004). However, the overall literature on narrative vividness is largely inconclusive as a result of how the concept has been defined. The seminal work on vividness is a chapter by Nisbett and Ross (1980) on the subject, which described vivid messages as those that are emotionally interesting, concrete (i.e., specific), imagery-provoking, and sensorially, temporally, or spatially proximate (p. 45). Though this definition served as the basis for a significant amount of subsequent work, Dillard (2014) highlighted a number of issues. Most notably, emotional interest and the provocation of imagery are outcomes of messages rather than features of them. As such, studies that have used this definition may have failed to gauge the persuasive power of vividness as an exclusive feature of messages.

To avoid this issue, we define narrative vividness independent of the outcomes that messages produce or their interdependence with audiences at which messages are aimed. Specifically, and similar to Keller and Block (1997), we define vividness as the degree to which a message is specific about individuals, their actions, the contexts in which those actions occur, and the actions’ outcomes in the story. In an illustrative example, an experimental manipulation by Smith and Schaffer (2000) contrasted “an increase in the probability of an accident” (nonvivid condition) with “a high risk of bloody, bone-crushing accidents” (vivid condition; p. 777).

Although other features of messages have also received significant attention, we focus on vividness because of its widespread presence in terrorist narrative propaganda (Braddock, 2012) and its likely associations with the components of the Dark Tetrad.

#### Terrorist Use of Narratives

Horgan (2014) rightly observed that analysts typically describe terrorist behavior as somehow “special.” Although terrorism is statistically and normatively irregular, there is no evidence to suggest that the social and psychological dynamics that drive terrorism are different from those that underpin “normal” behavior (Braddock and Horgan, 2016). How terrorist groups persuade their audiences is, in principle, similar to how nonviolent groups persuade theirs. Message content differs, but the psychological mechanisms that induce persuasion are essentially the same. When a communicator wishes to influence an audience, he/she seeks to align the audience’s beliefs, attitudes, and intentions with his/her goals. When these goals relate to the adoption of an extremist ideology that supports the use of violence, changes in beliefs and attitudes are commonly dubbed radicalization (Braddock, 2020).

Although radicalization remains a poorly defined term, it is most commonly used to describe a process of social and psychological change preceding an individual’s engagement in terrorism (see Horgan, 2014). However, some researchers contend that engagement in violent activity can occur before belief and attitude change (e.g., Sageman, 2008). In these cases, fighters retroactively adopt extremist ideologies to justify the violent action in which they have already engaged. Case analyses from terrorism studies support both scenarios, but in the case of the former, the persuasive strategies employed by terrorist organizations are fundamental to the dissemination of ideas that promote belief and attitude change that precedes violence.

Terrorists use several strategies to communicate with audiences and promote the adoption of their ideologies. One the most popular is the distribution of narratives containing themes consistent with the group’s ideology (Braddock and Horgan, 2016). On the popular *Stormfront* online discussion forums, radical white nationalists post narratives related to fictional race wars, confrontations with members of other races, and the exploits of the heroes of white nationalism.^2^ The Animal Liberation Front (ALF), a group responsible for violent attacks against people and property viewed as enemies of animal rights, maintains an online archive of sympathetic narratives created by ideological adherents.^3^ Several jihadist groups tell the story of Muhammad’s victory over the Meccans in the Battle of al-Badr as a metaphor for the group’s own struggles (Furlow and Goodall, 2011). As these and other examples illustrate, there is no shortage of terrorist groups using narratives to interact with audiences, particularly as it relates to imparting values that render individuals more likely to engage in violence (e.g., Atran, 2020, 2021).

Research on terrorist communication has shown that these narratives are effective vehicles for promoting the assimilation of extremist beliefs, attitudes, and intentions that can ultimately lead to engagement in violent behavior (e.g., Braddock, 2015). Taken together, the persuasive potency of narratives (Braddock and Dillard, 2016) and the ubiquity of terrorist narratives that prompt psychological processes that contribute to radicalization (Corman, 2011; Furlow and Goodall, 2011) suggest that “exposure to terrorist narratives can at least theoretically increase an individual’s risk for supporting terrorism” (Braddock and Horgan, 2016, p. 385).

Despite their persuasive power, it would be unwise to presume that an individual would adopt extremist beliefs or attitudes *solely* as a function of their exposure to a terrorist narrative. Indeed, the low base-rate of terrorist activity suggests that most who are exposed to terrorist narratives are not swayed by them. Moreover, meta-analyses of Braddock and Dillard (2016) suggested the presence of yet-unidentified moderators that can affect a narrative’s persuasiveness. These findings suggest that other factors—including personality factors—can affect an individual’s responses to persuasive narratives.

### The Dark Tetrad and Message Features

The Dark Tetrad is a collection of four personality traits that are linked to harmful outcomes. Initially, three “dark” personality traits—*narcissism*, *Machiavellianism*, and *subclinical psychopathy*—were summarized as the “Dark Triad” (Paulhus and Williams, 2002; Jakobwitz and Egan, 2006). Later researchers added *everyday sadism* to the taxonomy, bringing it to its current form (Chabrol et al., 2009).

The Dark Tetrad traits have been associated with aversive behavioral outcomes, including bullying (Baughman et al., 2012), juvenile delinquency (Chabrol et al., 2009), racist attitudes (Jones, 2013), and criminal activity (Hare and Neumann, 2008). Early research also attempted to directly link some of these traits with engagement in terrorism (e.g., Cooper, 1976; Hacker, 1976), though this work was largely dismissed as empirically and methodologically unsound (Horgan, 2005; Victoroff, 2005).

Although the components of the Dark Tetrad have “distinctive theoretical roots” (Jones and Paulhus, 2014, p. 28), their unique conceptualization and effects on behavioral outcomes are not always clear. Some have even argued that narcissism, Machiavellianism, and psychopathy are essentially interchangeable in non-clinical populations (e.g., McHoskey et al., 1998). However, research spearheaded by Daniel Jones and Delroy Paulhus has delineated the various aversive personalities (see Paulhus and Williams, 2002; Jones and Paulhus, 2009, 2010, 2011, 2014; Jones and Figueredo, 2012; Furnham et al., 2013). Notably, they have shown that the personality traits comprising the Dark Tetrad are often associated with the manifestation of different respective behavioral effects under different conditions. For instance, narcissism and subclinical psychopathy are both associated with aggressive behaviors, but manifest in response to different types of threats (Jones and Paulhus, 2010).

To explore how narcissism, Machiavellianism, subclinical psychopathy, and everyday sadism moderate the effects of features of narratives, it is important to first review the nature of these personality traits as well as their theorized association with support for terrorism.

#### Narcissism

Narcissism is defined by an internal conflict between “grandiose identity and underlying insecurity” (Jones and Paulhus, 2014, p. 29). Narcissists tend to have exalted views of themselves that are difficult to affirm (Paulhus and John, 1998). At the same time, these self-perceptions are often unstable, so confirming them is critical for the narcissist’s psychological well-being (Jordan et al., 2003). Given this need to validate their self-perceptions, narcissists perpetually seek to reinforce their own egos, which can lead to self-destructive behaviors (Morf and Rhodewalt, 2001; Vazire and Funder, 2006). This quest for grandiosity can be so strong as to promote a sense of entitlement that can manifest as hostility or aggression if that grandiosity is questioned or threatened (Bushman and Baumeister, 1998; Jones and Paulhus, 2010).

Given the link between narcissism and aggressive tendencies, early terrorism researchers claimed that terrorists tend to have narcissistic qualities (Lasch, 1979; Post, 1984, 1986; Pearlstein, 1991; Johnson and Feldmann, 1992). As outlined above, however, these models were often simplistic and were dismissed as the study of terrorism progressed. Recently, however, narcissism has been examined in conjunction with other factors that may predict an individual’s violent radicalization or engagement in terrorism. For instance, psychiatrists and other experts concluded that Anders Breivik, who killed 77 civilians in Norway in 2011, harbored a narcissistic personality that was exacerbated by delusions and the cultivation of extreme right-wing attitudes toward what he called “cultural Marxism” in Europe (Aspaas and Tørrissen, 2012; Jacobsen and Maier-Katkin, 2015).

Others have linked narcissism with sensation-seeking and risk-taking (e.g., Vazire and Funder, 2006), which Lankford (2013) argued are typical among terrorists that seek to conduct the most “daring and elaborate” attacks (e.g., suicide terrorism, p. 147). Some researchers have uncovered even more direct links between sensation-seeking and one’s predilection for extremist activity.

In summary, narcissism may not predict engagement in terrorism in and of itself. However, it is possible that narcissists are more prone to persuasion *via* terrorist narratives than non-narcissists. A significant proportion of terrorist propaganda is designed to illustrate how supporting the terrorist organization can fulfill an individual’s need to be part of something greater than him/herself. In doing so, terrorist propaganda may promise an individual the means to continuously reinforce his/her grand self-perceptions. As such, terrorist narratives may appeal to narcissists because they not only explain that a target recruit *can* be great, but also *how* that greatness can be achieved.

Given this, we offer the following hypothesis:

*H1*: Narcissism moderates the effect of exposure to terrorist narratives on beliefs, attitudes, and behavioral intentions, such that the persuasiveness of terrorist narrative content is greater for individuals who report higher levels of narcissism.

#### Machiavellianism

*Machiavellianism* is commonly characterized as a predisposition to regard other individuals as tools to be manipulated (Sutton and Keogh, 2000). Machiavellians tended to exploit and behave coldly toward others (Christie and Geis, 1970) and maintain a general cynicism and lack of morality in response to the world around them (Furnham et al., 2013). Jones and Paulhus (2009) added that Machiavellianism is also characterized by planning, coalition forming, and the maintenance of one’s reputation. This latter addition distinguishes Machiavellianism from subclinical psychopathy (see below). Whereas psychopaths tend to behave impulsively, abandon valued others, and have little regard for their reputations (Hare and Neumann, 2008; Jones and Paulhus, 2014), Machiavellians are careful to plan their behaviors such that they are simultaneously self-serving and reputation building. To illustrate, Machiavellians rarely fake weakness or manipulate those close to them (Shepperd and Socherman, 1997; Barber, 1998). Though these behaviors may serve their short-term interests, Machiavellians would avoid the resulting damage to their reputations. As summarized by Jones and Paulhus (2014), Machiavellianism is fundamentally characterized by a proclivity toward manipulation, callous affect, and an orientation for strategic calculation.

Machiavellian characteristics would seem to lend themselves to an understanding of terrorist motivation. After all, terrorists are often described as manipulative, callous, and strategically oriented. To our knowledge, however, no research has explored how Machiavellianism may affect the likelihood of an individual’s violent radicalization and/or engagement in terrorism.

The relative absence of work on Machiavellianism and terrorism begs the question as to why it should be included in empirical models predicting belief, attitude, or intention change in response to terrorist propaganda. We believe that Machiavellianism may moderate the effect of exposure to terrorist narratives for two reasons. First, terrorist propaganda often makes appeals to self-interest and reputation-building. Although terrorist narratives typically highlight the benefits of engagement to the group’s purported constituents, they also emphasize benefits that could befall the message’s target (Christien, 2016; Braddock, 2020). Second, terrorist propaganda can be particularly graphic in its depiction of enemy treatment (e.g., execution videos). The brutality of these messages may appeal to the callous nature of Machiavellians.

Therefore, we predict:

*H2*: Machiavellianism moderates the effect of exposure to terrorist narratives on beliefs, attitudes, and behavioral intentions, such that the persuasiveness of terrorist narrative content is greater for individuals who report higher levels of Machiavellianism.

#### Subclinical Psychopathy

Original descriptions of psychopathy tended to frame it as a severe personality disorder characterized by callousness and a lack of emotion (Cleckley, 1976). However, some experts (e.g., Ray and Ray, 1982) predicted that the study of psychopathy could extend beyond the clinical sphere to the mainstream (Furnham et al., 2013). Consistent with this conjecture, researchers began to treat psychopathy not as a categorical entity of mental illness, but as a component of an individual’s personality (see Levenson, 1992). Though this latter conceptualization, referred to as *subclinical psychopathy*, is associated with a “lighter” form of antisocial tendencies, it is nonetheless viewed as the most malicious of the original Dark Triad (Rauthmann, 2012). Subclinical psychopathy typically manifests as impulsivity or thrill-seeking coupled with a lack of empathy for others and a lack of remorse for one’s actions (Lilienfeld and Andrews, 1996; Kapoor, 2015).

Subclinical psychopathic characteristics likely interact with certain features of terrorist messages to influence their persuasiveness. For instance, individuals with a proclivity for sensation-seeking (of which thrill-seeking is a constituent factor; Zuckerman, 1971) tend to be drawn to messages that are presented in a dramatic, intense fashion (Donohew et al., 1991; Stephenson, 2003). Given this, it may be that terrorist narratives (which often depict dramatic, intense scenes related to the group’s conflict) will be more persuasive to those with a stronger expression of subclinical psychopathy. Moreover, sensation-seekers and impulsive individuals tend to seek out and engage in risky behaviors (Voigt et al., 2009; Braddock et al., 2011). Given the inherent risks associated with engagement in terrorism (i.e., capture, arrest, and death), terrorist narratives are likely to appeal to those that seek these risky thrills.

Given these arguments, we predict:

*H3*: Subclinical psychopathy moderates the effect of exposure to terrorist narrative on beliefs, attitudes, and behavioral intentions, such that the persuasiveness of terrorist narrative content is greater for individuals who report higher levels of subclinical psychopathy.

#### Everyday Sadism

Whereas most individuals feel upset after causing harm to an innocent person, others experience pleasure, excitement, or arousal. Rather than assuage the suffering of those around them, these individuals often pursue opportunities for brutality or cruelty (Baumeister and Campbell, 1999; Buckels et al., 2013). Most studies on this kind of disposition have focused on drastic forms of cruelty, like sexual aggression and violence (e.g., Federoff, 2008; Nietschke et al., 2009). However, Buckels et al. (2013) rightfully argue that some non-clinical individuals also enjoy other forms of cruelty, as evidenced by the popularity of violent media and sports, as well as the pervasiveness of police and military brutality (p. 2201). The authors argue that this enjoyment represents a less-extreme form of sadism which they refer to as *everyday sadism*.

In contrast to the other components of the Dark Tetrad, there has been very little empirical work on everyday sadism and its outcomes. In one study, Chabrol et al. (2009) demonstrated that sadism was significantly associated with antisocial behavior independently of narcissism, Machiavellianism, and subclinical psychopathy. Subsequently, everyday sadism was added to the factors of the Dark Triad to produce the Dark Tetrad. Moreover, individuals who scored high on a scale of implicit sadism were more prone to unprovoked aggression than their non-sadistic counterparts (Reidy et al., 2011). Finally, Buckels et al. (2013) found that relative to non-sadists, sadists were more likely to enjoy killing insects, aggress against innocent others, and intensify their attacks when they realize the target of their aggression would not retaliate.

To our knowledge, there has been no work on the effect of everyday sadism on violent radicalization. Popular media often characterize terrorists as sadists, motivated exclusively by an enjoyment of doling out pain. Although contemporary researchers have largely cast aside these overly simplistic models for explaining terrorism, it is possible that an audience member’s expression of everyday sadism influences the effect of terrorist narratives on violent radicalization.

As such, we predict:

*H4*: Everyday sadism moderates the effect of exposure to terrorist narratives on beliefs, attitudes, and behavioral intentions, such that the persuasiveness of terrorist narrative content is greater for individuals who report higher levels of everyday sadism.

### The Moderating Effect of Narrative Vividness

#### Vividness and Narcissism

As outlined above, narcissism is associated with sensation-seeking and risk-taking (Vazire and Funder, 2006). Moreover, high sensation-seekers are more likely to be persuaded by messages that are presented in a graphic, intense manner than their low sensation-seeking counterparts (Donohew et al., 1991; Stephenson, 2003). Zmigrod and Goldenberg (2021) additionally argued that when high sensation-seekers are unable to adapt their behaviors to changing environments and task demands, they are at greater risk for supporting ideologically inspired violence. Taken in concert, these findings suggest that the degree to which a terrorist narrative is comprised of language that depicts graphic scenes will moderate the extent to which narcissism influences the persuasiveness of that narrative.

So, we offer the following hypothesis:

*H5*: Narcissism moderates the effect of narrative vividness on beliefs, attitudes, and behavioral intentions, such that vivid narratives are more persuasive for individuals who report higher levels of narcissism.

#### Vividness and Machiavellianism

Recall that Machiavellians tend to be callous in nature. This cold-heartedness may render them more susceptible to persuasion *via* messages that include graphic descriptions of events. As a result, it is possible that the vividness of terrorist narrative propaganda influences the degree to which that propaganda affects a Machiavellian’s beliefs, attitudes, or intentions in relation to the terrorist organization.

To test this possibility, we test the following hypothesis:

*H6*: Machiavellianism moderates the effect of narrative vividness on beliefs, attitudes, and behavioral intentions, such that vivid narratives are more persuasive for individuals who report higher levels of Machiavellianism.

#### Vividness and Subclinical Psychopathy

Given the subclinical psychopath’s relative lack of empathy (Lilienfeld and Andrews, 1996), as well as the close association between subclinical psychopathy and Machiavellianism (which is characterized by callousness; Fehr et al., 1992), the graphic nature of vivid terrorist narrative propaganda may be more appealing to those with subclinical psychopathic tendencies. This may be particularly true if the narrative depicts violent treatment of enemies in a graphic way.

Following from this, we predict that:

*H7*: Subclinical psychopathy moderates the effect of narrative vividness on beliefs, attitudes, and behavioral intentions, such that vivid narratives are more persuasive for individuals who report higher levels of subclinical psychopathy.

#### Vividness and Everyday Sadism

Terrorist narratives often depict explicit acts of violence against purported enemies of the group in great detail. Consider, for example, the propaganda videos released by the so-called Islamic State (IS) that showed the immolation execution of a Jordanian pilot. They often also depict how the group’s enemies attacked individuals that the group purports to represent and defend. Hamas, for example, often includes stories on its website describing how the Israeli army has killed civilians in the Palestinian territories. The detail associated with depictions like these may provide everyday sadists with feelings of pleasure or excitement that render them more likely to be persuaded by the narrative.

Given this possibility, we believe that:

*H8*: Everyday sadism moderates the effect of narrative vividness on beliefs, attitudes, and behavioral intentions, such that vivid narratives are more persuasive for individuals who report higher levels of everyday sadism.

## Materials and Methods

### Participants

Data were gathered from a national, opt-in online survey panel of American adults through Qualtrics panels in the summer of 2018. Screening questions disqualified participants who were younger than 18 years old or could not understand English (the language in which all experimental materials were presented). After removing participants who completed the survey in less than 25% of the median completion time, provided non-differentiated data (also known as straight-lining), or failed to provide data altogether, the final sample comprised 268 participants. Respondent ages ranged from 18 to 76 years old (*M*_age_ = 33.24, *SD*_age_ = 11.91) and 79.1% of participants were male. A sensitivity analysis revealed the sample size (*N* = 268) to be sufficient for detecting small-to-medium effect sizes (critical *f*^2^ = 0.076) assuming statistical power of 0.80 and an alpha level of 0.05.

### Design and Procedure

We employed a posttest-only, between-subjects experimental design with two experimental conditions and one control condition. Independent variables included both individual-level (i.e., Dark Tetrad trait characteristics) and message-level (i.e., vividness) measures. Dependent variables were participant beliefs, attitudes, and intentions related to a group described in the stimuli and the violent actions it undertakes.

After enrolling in the study, respondents were directed to the survey website where they provided consent to participate. They were next directed to a screen welcoming them to the study and describing the nature of their participation. Participants were then prompted to click “Next” to move into the study.

Following the provision of consent, participants were directed to survey items measuring the Dark Tetrad trait characteristics, as well as other personality measures to be included in the analyses as moderators. Following the administration of these measures, participants were randomly assigned to one of three conditions in which they were exposed to a high-vividness narrative (*n* = 86), a low-vividness narrative (*n* = 90), or a control condition in which they were only informed of the group’s goals and actions (i.e., no narrative stimulus; *n* = 92). Participants then responded to survey items measuring their beliefs, attitudes, and intentions regarding the extremist group. They were then thanked for their participation and debriefed.

### Materials

All conditions began with a brief description of a group called the Homeland Liberation Alliance (HLA).^4^ This brief passage describes how the HLA launches occasional attacks into a neighboring country as part of ongoing hostilities between the HLA and the people of that country. This description also highlights the HLA’s grievances with the neighboring country, including the theft of its land, the decimation of its economy, and the country’s use of violence against the civilians in the group’s territory.

The narratives in the experimental conditions were adapted from a story that appeared on the website for the Islamic militant group, Hamas. This story describes an alleged incident in which a guard at an Israeli checkpoint executes a 74-year-old Palestinian woman after giving her water and posing for a photo. In the nonvivid condition, the account of the incident is presented in a straightforward manner and with little descriptive elaboration (e.g., *…the woman had already been dead for hours*). The narrative in the vivid condition featured much more detailed language, including graphic descriptions of the shooting itself (e.g., *…the woman had died hours before, her blood having drained out onto the street where it had dried to a dark brown gel*). In the control condition, participants were exposed only to the brief introductory description of the group, its grievances, and its goals.

The vivid and nonvivid narrative conditions were comparable in length, dialectical complexity (complexity achieved through the inclusion of multiple viewpoints), and integrative complexity (complexity achieved with the use of multifaceted arguments; see Baker-Brown et al., 1990). The control condition contained no narrative content and was thus shorter than the passages in the narrative conditions.

Both narrative stimuli and the control condition are available *via* the Open Science Framework at https://tinyurl.com/darktetradconditions.

### Measures

All scales used for data collection are available for review at the Open Science Framework at https://tinyurl.com/darktetradscales.

#### The Dark Tetrad

##### Narcissism, Machiavellianism, and Subclinical Psychopathy

To measure narcissism, Machiavellianism, and subclinical psychopathy, we used Short Dark Triad (SD3) of Jones and Paulhus (2014). For each of the three subscales, participants were asked to rate their agreement on a Likert-type scale ranging from 1 (Strongly disagree) to 7 (Strongly agree). Sample items include *I insist on getting the respect I deserve* (narcissism), *most people can be manipulated* (Machiavellianism), and *payback needs to be quick and nasty* (subclinical psychopathy).

We used the SD3 for two key reasons. First, the brevity of the SD3 avoids validity issues related to participant fatigue. Although other measures of narcissism (the shortened Narcissistic Personality Inventory; Ames et al., 2006), Machiavellianism (the Mach-IV; Christie and Geis, 1970), and psychopathy (the Self-Report Psychopathy Scale; Williams et al., 2007 and Psychopathic Personality Inventory; Lilienfeld and Andrews, 1996) have been used extensively, none of the individual subscales has fewer than 16 items. To measure all three traits using these scales, a questionnaire would need to contain no fewer than 65 items. In contrast, the SD3 measures all three components of the Dark Triad with a total of 27 items.

Second, past comparisons of the SD3 to another shortened measure of the Dark Triad—Dirty Dozen of Jonason and Webster (2010) have favored the former over the latter (c.f., Lee et al., 2013). The Dirty Dozen has been criticized for its weak association to quintessential measures of the three traits (Miller et al., 2012) and the fact that the cross-correlations tend to be stronger than convergent correlations with gold-standard measures (Rauthmann, 2013; Jones and Paulhus, 2014). In contrast, the SD3’s subscales have demonstrated strong correlations with their respective standard measurement instruments (*r* = 0.82–0.92; Jones and Paulhus, 2014, p. 34). Given the shortcomings of other available measures of narcissism, Machiavellianism, and subclinical psychopathy, the SD3 represents the best option for measuring these three traits. Over multiple studies, Jones and Paulhus (2014) reported acceptable reliability estimates for the narcissism (average *ɑ* = 0.72), Machiavellianism (average *ɑ* = 0.76), and psychopathy (average *ɑ* = 0.75) subscales. Our analysis offered similar results, providing acceptable reliability estimates for the narcissism^5^ (*α* = 0.71), Machiavellianism (*α* = 0.82), and subclinical psychopathy subscales (*α* = 0.82).

##### Everyday Sadism

To measure everyday sadism, we administered the 10-item Short Sadistic Impulse Scale (SSIS; O’Meara et al., 2011). Psychometric analysis of the SSIS (O’Meara et al., 2011, *α* = 0.86), as well as studies that have employed it (e.g., Buckels et al., 2013, *α* = 0.87), have shown the scale to have good internal consistency. Although the original version of the SSIS was presented such that participants responded to all the items dichotomously (i.e., “like me” vs. “unlike me”), we adapted the SSIS such that it appeared as a series of Likert-type scales ranging from 1 (Strongly disagree) to 7 (Strongly agree). Questions on the scale included *I enjoy seeing people get hurt* and *I have hurt people because I could*. Scale reliability was good (*α* = 0.93).

#### Beliefs, Attitudes, and Intentions

Consistent with Fishbein and Ajzen’s reasoned action theory, as well as meta-analyses evaluating the persuasive effects of narratives (e.g., Braddock and Dillard, 2016), in this study, we consider beliefs, attitudes, and intentions to be outcomes indicative of greater persuasion by exposure to stimulus narratives. That is, higher scores on belief, attitude, and intention measures indicate greater persuasion by the passage to which participants are exposed. Details associated with these scales are outlined in the sections below.

##### Beliefs

Beliefs represent impartial judgments regarding that which we believe to be true and false about the world (Fishbein and Ajzen, 1975). Because they are unvalenced, they are not motivational; they are simply our perceptions of what goes on around us. As such, any measure of beliefs would gauge the degree to which an individual accepts a communicator’s account of real-world facts. Accordingly, participants reported the extent to which they agreed with seven items that represented facts contained within the narratives to which they were exposed (1 = strongly disagree) to (7 = Strongly agree). Principal component analysis with oblimin rotation showed that the items represented two correlated sub-scales that, respectively, measured beliefs about the HLA’s use of violence (e.g., *I believe that the HLA’s attacks are likely to lead to changes that the group hopes for*; *α* = 0.76) and beliefs about the HLA’s enemies (e.g., *I believe that the neighboring country is stealing land from the HLA’s home territory*; *α* = 0.77) identified in the narrative. Both subscales were used as outcome variables.

##### Attitudes

In contrast to beliefs, attitudes are valenced judgments about a particular situation or behavior (Ajzen and Fishbein, 1967). Therefore, attitude measures should determine the degree to which participants agree with motivational assertions espoused by a communicator. To measure the extent to which the different narrative stimuli influenced participant attitudes, we asked respondents to indicate the degree to which they agreed with seven statements regarding the HLA’s activities and statements (e.g., *The neighboring country deserves to be attacked for what it does;* 1 = strongly disagree to 7 = strongly agree). These items loaded on two factors, with the second factor defined by a single item. Removal of the outlying item yielded a good reliability estimate for the scale comprising the remaining items (*α* = 0.88).

##### Behavioral Intentions

Intentions represent perceived motivations to engage in specific behaviors. To gauge participants’ intentions to act in support of the HLA, we presented them with a 10-item index (e.g., *If I lived in the HLA’s territory, I would consider using deadly weapons against the HLA’s enemies*). All items in the scale loaded on a single factor, the reliability estimate of which was good (*α* = 0.93).

#### Control Variables

In addition to the measures outlined above, we also measured several constructs that could be included in our analyses as controls, given the degree to which past research has shown them to influence outcomes peripheral to those examined in this study (e.g., risky health behaviors). These indices measured perceptions of general self-efficacy (Schwarzer and Jerusalem, 1995); impulsive sensation-seeking (Zuckerman, 1996); and the composite elements of the Big Five Inventory (see John and Srivastava, 1999): extraversion, agreeableness, conscientiousness, neuroticism, and openness to experience.

## Results

The pre-registration of all confirmatory analyses is available at: https://osf.io/hu6z9. All analyses were conducted using SPSS 25.0 and R 4.1.0.

### Descriptive Analyses

Table 1 presents the mean values, standard deviations, and bivariate correlations of all assessed variables. Variables that comprise the Dark Tetrad were moderately to highly positively associated. In addition, Dark Tetrad traits were significantly correlated with most outcome variables. Significant associations between the control variables and the dependent and moderator variables verified the rationale for including them in subsequent analyses as controls.

**Table 1:** Means, SDs, and bivariate correlations.

	*Variable*	*M*	*SD*	1	2	3	4	5	6	7	8	9	10	11	12	13	14	15	16
1	Narcissism	3.97	0.96	1															
2	Machiavellianism	4.23	1.03	0.36^**^	1														
3	Subcl. psychopathy	3.25	1.40	0.44^**^	0.66^**^	1													
4	Everyday sadism	2.50	1.49	0.31^**^	0.48^**^	0.74^**^	1												
5	Beliefs: Violence	4.05	1.29	0.19^**^	0.42^**^	0.38^**^	0.38^**^	1											
6	Beliefs: Invaders	4.50	1.24	0.14^**^	0.33^**^	0.18^**^	0.19^**^	0.51^**^	1										
7	Attitudes	4.39	1.25	0.12	0.42^**^	0.28^**^	0.27^**^	0.61^**^	0.64^**^	1									
8	Intention to support	4.02	1.31	0.27^**^	0.52^**^	0.41^**^	0.38^**^	0.53^**^	0.51^**^	0.75^**^	1								
9	Self-efficacy	4.35	0.94	0.24^**^	0.14^*^	−0.10	−0.15^*^	0.09	0.15^*^	0.17^**^	0.11	1							
10	Thrill seeking	3.05	0.76	0.47^**^	0.49^**^	0.59^**^	0.46^**^	0.28^**^	0.12	0.20^**^	0.33^**^	0.21^**^	1						
11	Imp. decision making	3.03	1.02	0.28^**^	0.26^**^	0.43^**^	0.37^**^	0.16^**^	0.07	0.11	0.21^**^	0.06	0.62^**^	1					
12	Extraversion	3.11	0.70	0.39^**^	0.01	0.09	0.03	0.05	−0.08	−0.07	0.00	0.34^**^	0.22^**^	0.16^**^	1				
13	Agreeableness	3.69	0.68	−0.12^*^	−0.35^**^	−0.56^**^	−0.62^**^	−0.17^**^	−0.07	−0.05	−0.23^**^	0.45^**^	−0.31^**^	−0.25^**^	0.17^**^	1			
14	Conscientiousness	3.61	0.69	−0.04	−0.22^**^	−0.47^**^	−0.49^**^	−0.11	−0.00	−0.04	−0.15^*^	0.51^**^	−0.33^**^	−0.34^**^	0.25^**^	0.71^**^	1		
15	Neuroticism	2.82	0.80	−0.02	0.28^**^	0.38^**^	0.36^**^	0.13^*^	0.11	0.09	0.16^**^	−0.32^**^	0.27^**^	0.27^**^	−0.39^**^	−0.58^**^	−0.64^**^	1	
16	Openness to experience	3.74	0.70	0.27^**^	0.18^**^	−0.03	−0.10	0.01	0.19^**^	0.12^*^	0.11	0.60^**^	0.27^**^	0.11	0.29^**^	0.34^**^	0.35^**^	−0.08	1

^*^p < 0.05;

** *p* < 0.01.

Deviating from the pre-registration, we first conduct two multivariate analyses of variance to determine main effects of the experimental manipulations. The Box test (*M* = 12.10, *p = 0*.294) and Levene test (0.106 < *p < 0*.950) emphasized that parametric tests could be computed. Findings indicated that there were no significant differences between the narrative and control groups with respect to how they influenced participants’ beliefs about invaders [*F*(1, 268) = 2.01, *p* = 0.16, *η^2^* = 0.01], beliefs about the use of violence [*F*(1, 268) = 1.89, *p* = 0.17, *η^2^* = 0.01], attitudes [*F*(1, 268) = 0.27, *p* = 0.61, *η^2^* = 0.00], or behavior intentions [*F*(1, 268) = 1.70, *p* = 0.19, *η^2^* = 0.01]. See Table 2 for a synopsis of the data associated with these analyses.

**Table 2:** Properties of study outcomes by narrative condition.

		Beliefs Invaders		Beliefs Violence		Attitudes		Intentions	
Condition	*n*	*M*	*SD*	*M*	*SD*	*M*	*SD*	*M*	*SD*
Narrative	176	4.52	1.29	4.05	1.25	4.38	1.32	4.00	1.34
Control	91	4.47	1.16	4.04	1.37	4.41	1.09	4.05	1.24

Moreover, there was no significant effect of narrative vividness on salient outcomes [Beliefs_Invaders_: *F*(1, 268) = 1.01, *p* = 0.37, *η^2^* = 0.01; Beliefs_Violence_: *F*(1, 268) = 0.95, *p* = 0.39, *η^2^* = 0.01; Attitudes: *F*(1, 268) = 0.37, *p* = 0.69, *η^2^* = 0.00; and Behavior intentions: *F*(1, 268) = 0.85, *p* = 0.43, *η^2^* = 0.01]. Descriptive data associated with these analyses are summarized in Table 3.

**Table 3:** Properties of study outcomes by narrative vividness.

		Beliefs Invaders		Beliefs Violence		Attitudes		Intentions	
Condition	*n*	*M*	*SD*	*M*	*SD*	*M*	*SD*	*M*	*SD*
Vivid	86	4.58	1.38	4.11	1.41	4.40	1.51	3.94	1.41
Nonvivid	90	4.46	1.19	4.01	1.10	4.36	1.13	4.06	1.28

Surprising though these findings were, our predictions focused on the interactions between narrative vividness, the Dark Tetrad, and narrative engagement. Moreover, the emergence of interaction effects would supersede any lack of a main effect for narrative condition or vividness. So, given the absence of these main effects, we turned to the moderation analyses that formed the basis of our hypotheses.

### Moderated Effects of Narrative Exposure

To assess Hypotheses 1–4, which predicted interaction effects between the Dark Tetrad traits and narrative exposure, we tested all moderation relationships simultaneously for all four outcome variables. Shapiro–Wilk tests highlighted that none of the dependent variables was normally distributed (Beliefs Invaders: *W* = 0.975, *p = 0*.000; Beliefs Violence: *W* = 0.984, *p* = 0.004; Attitudes: *W* = 0.981, *p* = 0.001; and Intentions: *W* = 0.969, *p* = 0.000). To respond to these failed assumption tests, all models were estimated using maximum likelihood estimation with robust (Huber-White) standard errors and a scaled test statistic that is (asymptotically) equal to the Yuan-Bentler test statistic (i.e., MLR estimate for incomplete data).

Specifically, we modeled a path analysis that included narcissism, Machiavellianism, subclinical psychopathy, and everyday sadism, as well as interaction terms between these traits and the experimental conditions (i.e., control vs. any narrative condition) as independent variables; both belief sub-scales, attitudes, and behavioral intentions were the respective dependent variables. Control variables, as well as age and gender, were modeled to predict each respective outcome. Residuals of all Dark Tetrad variables were proposed to be correlated. The model also estimates covariances between all dependent measures.

The aforementioned model did not achieve acceptable fit [*χ*^2^(56) = 372.77, *p* = 0.002, CFI = 0.75, RMSEA = 0.15, SRMR = 0.13]. Control variables, aside from gender and age, were removed from the model as they correlated with the target independent variables of the Dark Tetrad (i.e., the model was at risk of multicollinearity; see Table 1 for correlations). By removing these variables, the modified model achieved acceptable fit [*χ*^2^(28) = 54.34, *p* = 0.002, CFI = 0.97, RMSEA = 0.06, SRMR = 0.05; *R*^2^_BeliefsViol_ = 0.25, *R*^2^_BeliefsInv_ = 0.15, *R*^2^_Attitudes_ = 0.20, *R*^2^_Intentions_ = 0.31]. Table 4 reports the results of this analysis.

**Table 4:** Main and interaction effects of dark tetrad traits and narrative exposure on participant beliefs, attitudes, and intentions.

Variable	Beliefs Invaders		Beliefs Violence		Attitudes		Intentions	
	*β*	95% CI	*β*	95% CI	*β*	95% CI	*β*	95% CI
Sex	0.09	−0.24, 0.42	−0.07	−0.33, 0.19	0.04	−0.26, 0.34	−0.08	−0.38, 0.22
Age	0.01	−0.01, 0.02	0.01	−0.01, 0.03	0.00	−0.02, 0.02	0.00	−0.02, 0.02
Condition (narrative v. control)	0.02	−0.28, 0.31	−0.04	−0.34, 0.26	−0.08	−0.36, 0.20	−0.12	−0.35, 0.11
Narcissism	0.15^*^	0.01, 0.29	0.11	−0.05, 0.27	0.06	−0.10, 0.22	0.15^*^	0.00, 0.30
Machiavellianism	0.39^***^	0.17, 0.61	0.32^**^	0.10, 0.54	0.46^***^	0.24, 0.68	0.50^***^	0.29, 0.70
Subclinical psychopathy	−0.17	−0.37, 0.03	−0.01	−0.21, 0.19	−0.09	−0.29, 0.11	−0.03	−0.26, 0.20
Everyday sadism	0.12	−0.03, 0.28	0.20^**^	0.06, 0.34	0.12	−0.02, 0.26	0.14	−0.02, 0.30
Narcissism * Exposure	−0.03	−0.19, 0.13	−0.09	−0.26, 0.09	−0.10	−0.28, 0.08	−0.12	−0.30, 0.06
Machiavellianism * Exposure	0.12	−0.11, 0.36	0.28^*^	0.02, 0.54	0.22^**^	0.00, 0.44	0.14	−0.10, 0.34
Subclinical psychopathy * Exposure	−0.11	−0.37, 0.15	−0.15	−0.41, 0.11	0.03	−0.23, 0.29	0.01	−0.27, 0.29
Everyday sadism * Exposure	0.11	−0.11, 0.33	0.01	−0.19, 0.21	−0.07	−0.25, 0.11	0.06	−0.14, 0.26
*R* ^2^	0.15		0.25		0.20		0.31	

* *p* < 0.05;

** *p* < 0.01;

*** *p* < 0.001.

**Figure 1:**
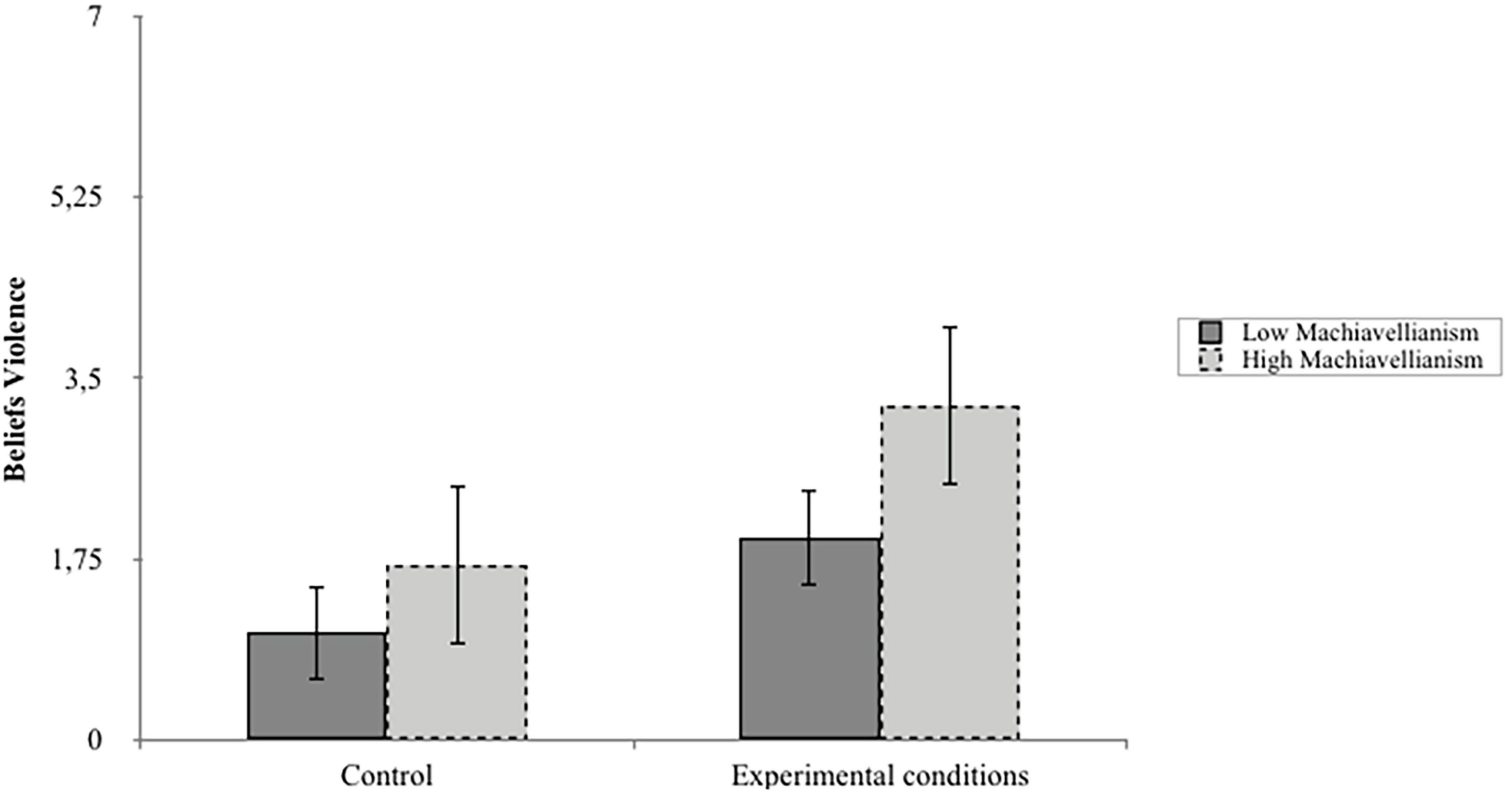
The moderating role of Machiavellianism by narrative exposure on Homeland Liberation Alliance (HLA) Use of Violence.

**Figure 2:**
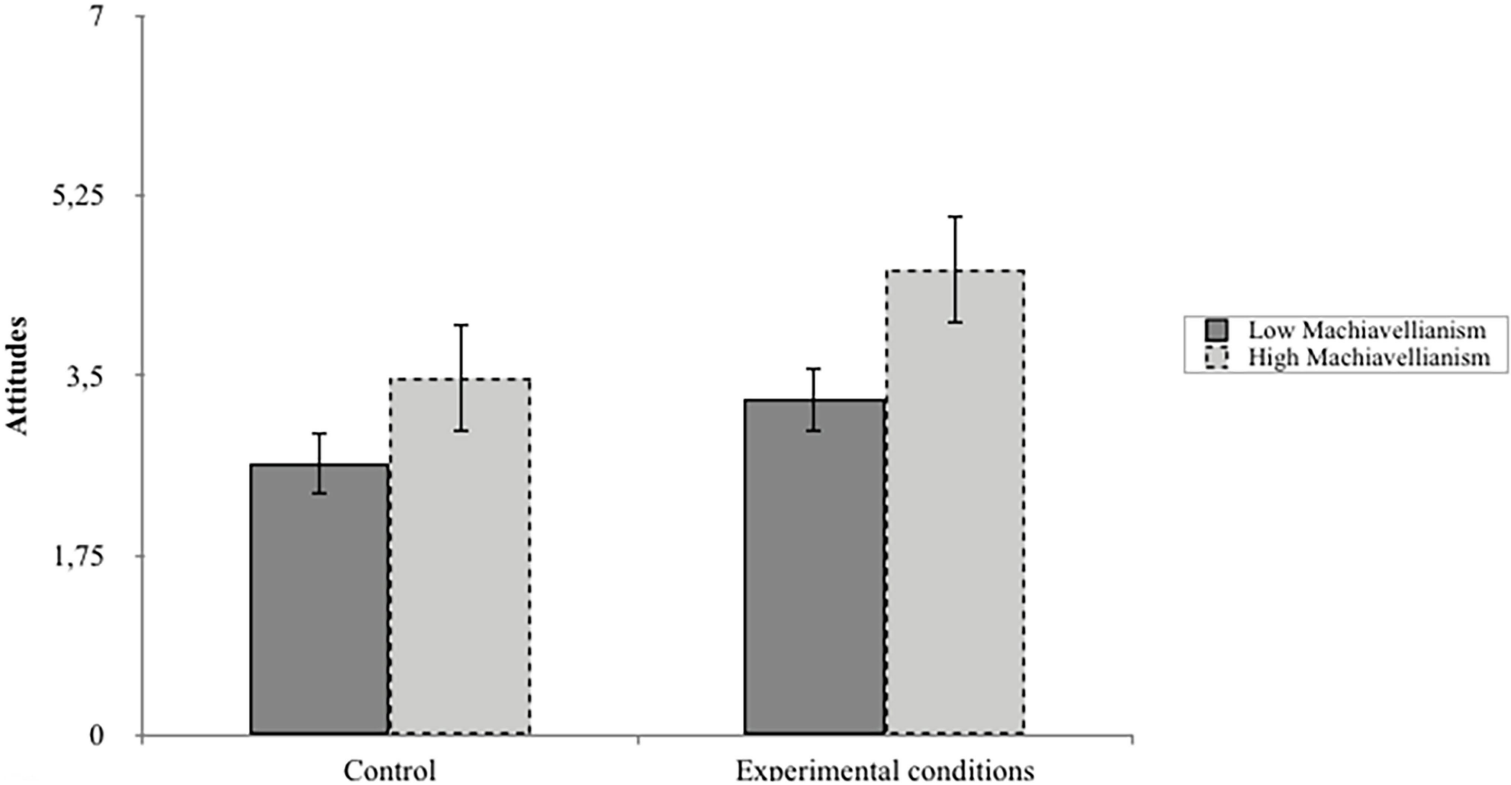
The moderating role of Machiavellianism by narrative exposure on Attitudes about the HLA.

These results show that Machiavellianism moderates the effect of narrative exposure on beliefs about the HLA’s use of violence and attitudes about the HLA. Simple slope analyses demonstrated that participants who reported higher levels of Machiavellianism (as divided by median split) found the HLA’s narratives to be more persuasive in terms of beliefs about the HLA’s use of violence and attitudes toward the HLA more generally than the control message (*m*_BeliefsViol_ = 1.54, *t* = 2.80, *p* < 0.01; *m*_Attitudes_ = 1.05, *t* = 2.01, *p* < 0.05; See Figures 1, 2). In addition, higher levels of Machiavellianism were associated with higher scores on all outcomes. These results suggest indirect and direct paths by which Machiavellianism can predict audience responses to terrorist narratives.

Moreover, narcissism had a significant positive direct effect on beliefs regarding the invaders in the HLA’s messages and their intentions to support the HLA. Everyday sadism similarly exerted a significant positive effect on participants’ beliefs about the HLA’s use of violence as a form of defense. Neither the effect of narcissism nor sadism were moderated by narrative exposure. Taken together, these results partially support H2, but fail to support H1, H3, or H4.

### Moderated Effects of Narrative Vividness

To examine Hypotheses 5–8, we replicated the first set of analyses, but included interaction terms of the Dark Tetrad traits with the experimental condition “narrative vividness.” The non-vivid narrative condition was treated as the control condition. As discussed before, we first estimated a model that specified relationships between the control variables and all four dependent variables. Once again, the model did not achieve acceptable fit [*χ*^2^(56) = 256.14, *p* = 0.002, CFI = 0.76, RMSEA = 0.14, SRMR = 0.12]. As in the first model, all control variables aside from gender and age were removed from the model given their potential multicollinearity with the Dark Tetrad variables. Removing the control variables improved model fit to an acceptable level [*χ*^2^(28) = 39.14, *p* = 0.079, CFI = 0.98, RMSEA = 0.05, SRMR = 0.04; *R*^2^_BeliefsViol_ = 0.36, *R*^2^_BeliefsInv_ = 0.20, *R*^2^_Attitudes_ = 0.28, *R*^2^_Intentions_ = 0.38].

**Figure 3:**
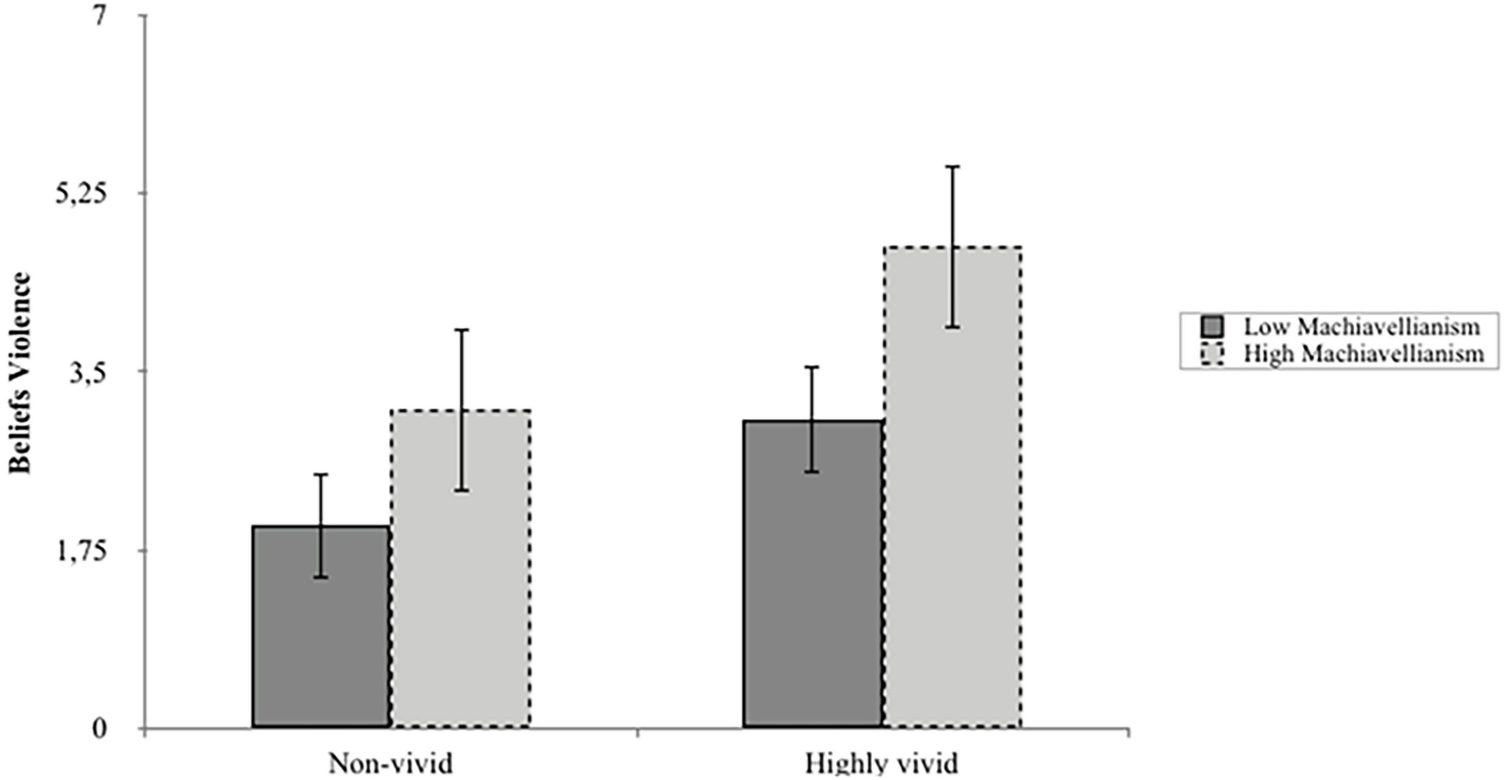
The moderating role of Machiavellianism by narrative vividness on HLA Use of Violence.

Results of this analysis are reported in Table 5 and Figure 3. Foremost, the results further highlighted the importance of Machiavellianism. Slope analyses demonstrated that when exposed to a vivid (relative to a non-vivid) narrative, individuals who rate high on Machiavellianism report beliefs consistent with the HLA’s use of violence (*m*_BeliefsViol_ = 1.61, *t* = 2.52, *p* < 0.05; see Figure 3). Results further demonstrated that Machiavellianism moderated the effect of narrative vividness on beliefs consistent with the HLA’s use of violence (see Table 5). Consistent with the first set of analyses, results also showed that Machiavellianism directly predicted higher levels of all outcomes.

**Table 5:** Main and interaction effects of dark tetrad traits and narrative vividness on participant beliefs, attitudes, and intentions.

*Variable*	*Beliefs Invaders*		*Beliefs Violence*		*Attitudes*		*Intentions*	
	*β*	95% CI	*β*	95% CI	*β*	95% CI	*β*	95% CI
Sex	0.21	−0.15, 0.57	0.13	−0.10, 0.37	0.12	−0.18, 0.42	0.03	−0.27, 0.33
Age	0.01	−0.01, 0.03	0.01	−0.01, 0.03	0.00	−0.02, 0.02	0.01	−0.01, 0.03
Condition (vivid v. nonvivid)	0.22	−0.12, 0.56	0.19	−0.11, 0.49	0.12	−0.22, 0.46	−0.02	−0.32, 0.28
Narcissism	0.08	−0.16, 0.32	0.03	−0.17, 0.23	−0.06	−0.30, 0.18	0.06	−0.14, 0.26
Machiavellianism	0.47^***^	0.21, 0.73	0.52^***^	0.30, 0.74	0.61^***^	0.37, 0.85	0.54^***^	0.32, 0.76
Subclinical psychopathy	−0.21	−0.47, 0.05	−0.04	−0.30, 0.22	−0.01	−0.27, 0.25	0.03	−0.29, 0.35
Everyday sadism	0.20^*^	0.00, 0.40	0.20^*^	0.00, 0.40	0.07	−0.13, 0.27	0.16	−0.08, 0.40
Narcissism ^*^ Vividness	−0.21	−0.51, 0.09	−0.18	−0.42, 0.06	−0.29	−0.61, 0.03	−0.08	−0.34, 0.18
Machiavellianism ^*^ Vividness	0.05	−0.23, 0.33	0.31^**^	0.07, 0.55	0.17	−0.09, 0.43	−0.10	−0.32, 0.12
Subclinical psychopathy ^*^ Vividness	−0.08	−0.46, 0.30	−0.18	−0.54, 0.18	−0.14	−0.50, 0.22	−0.12	−0.53, 0.29
Everyday sadism ^*^ Vividness	0.13	−0.17, 0.43	0.08	−0.20, 0.31	0.14	−0.16, 0.44	0.30	−0.04, 0.64
*R* ^2^	0.20		0.36		0.28		0.38	

* *p* < 0.05;

** *p* < 0.01;

*** *p* < 0.001.

Although everyday sadism did not interact with narrative vividness to predict any outcomes, it did directly predict participant beliefs about the HLA’s use of violence and the group’s enemies in this analysis.

Taken together, these results offer partial support for H6, but not for H5, H7, or H8.

## Discussion

### Summary of Results

The goal of this study was to determine whether the traits of the Dark Tetrad moderate the effect of different kinds of terrorist narrative stimuli to enhance their persuasive effects. Results indicated that this prediction was largely limited to Machiavellianism. Neither narcissism, subclinical psychopathy, nor everyday sadism moderated the effects of narrative exposure or narrative vividness on persuasive outcomes related to support for the source of terrorist propaganda.

These results highlight the risk in assuming that any form of terrorist propaganda will be universally persuasive. Our findings support the long-held notion that the effectiveness of persuasive messages depends, at least in part, on individual characteristics (Bouhana, 2019). In the next section, we turn to the complexities associated with these findings, as well as their implications for understanding the persuasiveness of terrorist propaganda.

### The Appeal of Terrorist Narratives to Machiavellians

Our results showed that those who scored high on the Machiavellianism scale (high-Machs) reported beliefs, attitudes, and intentions consistent with the goals of the terrorist organization, regardless of whether those goals were presented narratively. The interaction between Machiavellianism and narrative exposure also significantly predicted beliefs about the terrorist group’s use of violence and attitudes about the terrorist group itself. This effect was even more pronounced when accounting for propaganda vividness, as high-Machs who were exposed to *vivid* narrative propaganda reported beliefs consistent with the terrorist group’s use of violence to a significantly greater degree than their low-Mach counterparts. Taken together, these results suggest that Machiavellianism is a key trait within the Dark Tetrad that can shape processing of terrorist narratives.

This was a somewhat surprising finding given that other elements of the Dark Tetrad—subclinical psychopathy and everyday sadism—have typically been more associated with aggression than Machiavellianism (see Mededovic and Petrovic, 2015; Paulhus et al., 2021). However, a closer consideration of Machiavellianism, its correlates, and how they affect goal-directed behavior sheds some light on the result. The association between Machiavellianism and alexithymia, a failure to recognize or understand one’s own emotions or how one’s behavior might affect others’ emotions, may provide some explanation.

Alexithymia relates to a person’s tendency to experience their emotions “shallowly,” reducing their motivation to act in response to those emotions (see Wagner and Lee, 2008). In the context of the current study, Machiavellianism’s direct and indirect effects on persuadability by HLA narrative propaganda may be explained by alexithymia. Specifically, high-Machs may be less able to recognize negative emotions (like guilt or shame) that might result from supporting the views of an extremist group or considering the actions they might undertake on behalf of that group. For instance, in considering the HLA’s violent activities, high-Machs may have been less able to perceive vicarious guilt that would normally result from envisioning engaging in violent activities to support the HLA. Given their relative inability to perceive these negative emotions, they may have been more amenable to expressing support for the worldview espoused by the HLA and the group’s strategic use of violence. In short, Machiavellians may have been unable to emotionally process the negative implications of the HLA’s activities (or their own engagement in those activities), making them less likely to resist persuasion by the narrative propaganda espoused by the group.

Taken together, the results suggest that Machiavellianism enhances susceptibility to messages espoused *via* terrorist narratives. This persuasive susceptibility renders Machiavellians more vulnerable to adopting ideas consistent with vivid terrorist narratives. This fits with many models of radicalization that describe the process as incremental social and psychological change whereby an individual comes to support the use of terrorism (or in some cases, engage in violence themselves; see Horgan, 2014). In this way, Machiavellians may be more prone to radicalization due to persuasion by vivid terrorist narratives.

### The Failure of Narcissism, Subclinical Psychopathy, and Everyday Sadism to Moderate the Effects of Narrative Exposure or Vividness

Contrary to expectations, narcissism did not moderate the effect of narrative exposure or narrative vividness on the persuasiveness of the HLA propaganda. Recall that narcissists are characterized by grandiose perceptions of self-worth combined with an insatiable need for reinforcement of their unique qualities. When presented with messages that advocate becoming part of something greater than oneself (as much terrorist propaganda does), narcissists may be averse to the prospect of joining a group that does not feature him/her as the central figure. Indeed, some studies have shown that narcissists are primarily attracted to group membership only when they can rise to leadership (Zitek and Jordan, 2016) or when their joining the group is met with an individual reward (Nevicka et al., 2011). In this way, a narcissist’s quest for grandiosity may conflict with the idea of engaging in risky behaviors on behalf of a group when those behaviors are not promoted as personally beneficial.

This may be the case in the current study. The stimulus to which participants were exposed and the questions to which they responded did not mention personal gain. Instead, all outcomes were related to perceptions of the HLA *as a group* and the individual’s intention to support it. This finding suggests that narcissism is unlikely to have an effect on the persuasiveness of terrorist narrative propaganda when that propaganda does not mention *individual-based* rewards or opportunities for *individual* glory.

Subclinical psychopathy similarly failed to moderate the relationship between narrative exposure or narrative vividness and persuasion. Though this finding was also unexpected, research on the role of psychopathy in one’s preference for group membership and/or group efficacy can be instructive. For instance, Baysinger et al. (2014) demonstrated that when psychopathy is treated as a continuum, it is inversely related to one’s perceptions of a group and positively related to group dysfunction. O’Neill and Allen (2014) similarly showed psychopathy to be positively associated with intra-group conflict. Bell (2007) also highlighted that psychopathy was negatively related to conflict resolution and overall group performance. Taken together, the literature indicates that like narcissists (though for different reasons), subclinical psychopaths may be averse to membership in groups that recruit them.

Finally, everyday sadism failed to interact with narrative exposure or vividness to predict the persuasiveness of the narrative stimulus. This non-significant finding was perhaps the most surprising, given that (a) sadists tend to enjoy violent material more than non-sadists (Baumeister and Campbell, 1999; Buckels et al., 2013) and (b) the vividness of the propaganda varied primarily by the degree to which it was graphic in its portrayal of violence. Although this result was surprising, past work on sadism and its psychological correlates may provide some insight into the counterintuitive result. Specifically, past evidence indicates that sadistic tendencies are inversely related to empathy (Erickson and Sagarin, 2021), suggesting that everyday sadists would be less capable of emotionally understanding the tribulations of the HLA’s constituents depicted in the stimulus propaganda. As such, it is possible that everyday sadists may have *enjoyed* visualizing the violent scenes depicted in the stimulus narratives; but, without the ability to empathize with the HLA and its constituents, everyday sadists were not sufficiently motivated to adopt beliefs, attitudes, or intentions consistent with their propaganda.

### Practical Implications for Preventing Radicalization

In addition to expanding our conceptual understanding of terrorist narratives, the role of personality traits, and the combined effect of both, the findings reported in the current study also offer practical insight for contending with challenges posed by the persuasiveness of terrorist propaganda.

Most notably, our results suggest that Machiavellianism enhances the persuasiveness of narrative terrorist propaganda, particularly when that propaganda features vivid descriptions of narrative events. It is, therefore, critical to identify the kinds of media messages preferred by Machiavellians to (a) recognize when terrorist propaganda may appeal to them, and (b) develop effective counter-messages intended to neutralize the persuasive effects of all kinds of terrorist propaganda. Past work on the Dark Triad and psychological correlates that predict media use has shown that Machiavellianism is strongly related with sensation-seeking (Lu, 2008; Dickey, 2014). For its part, sensation-seeking has been empirically linked to a preference for content that is novel and unpredictable, and arouses sensory and affective responses (Donohew et al., 1991; Stephenson and Southwell, 2006; Wang et al., 2015).

Identification of these links allows us to better understand and target high-Machs. Specifically, in developing narrative content intended to challenge terrorist narrative propaganda (see Braddock and Horgan, 2016), it may be useful to imbue that content with vivid descriptions of narrative events to arouse sensory and affective responses that reduce the appeal of terrorist narratives. In this way, developers of narratives intended to challenge terrorist narrative propaganda can leverage the sensation-seeking tendencies of Machiavellians to render their counter-narratives more persuasive.

Despite the practical utility of targeting high-Machs with messages that are tailored to undermine the persuasive appeal of terrorist narratives, it may be difficult to identify and isolate these audiences without a nuanced analysis that gauges the personality dispositions of audiences. To the degree that future research is successful in identifying methods for doing so, counter-messages targeting narcissists and high-Machs can be better constructed to resonate with their intended audiences.

### Limitations and Future Research

Although the present study has implications for our understanding of how terrorist propaganda can influence its intended audiences, our findings are qualified by some limitations that can also be addressed in future research. First, we used only a single, text-based stimulus in testing the moderating effects of narrative exposure and vividness on propaganda persuasiveness. The use of this stimulus provides preliminary insight, but future work can expand this understanding through the use of stimuli characterized by different ideological focus and presented using different media.

The subtle differences in how video-based narratives are processed relative to text (see Shen et al., 2015; Braddock and Dillard, 2016), coupled with the fact that most terrorist narratives are consumed *via* engagement with content on interactive digital media (Ashraf, 2021), demands that future work in this domain evaluate potential interactions between personality traits like the Dark Tetrad and video-based terrorist narrative processing to evaluate persuasive efficacy. For instance, personality traits that favor intense visual stimuli may respond differently under these conditions.

In a similar sense, future research should also evaluate the interactions between personality and narrative features using extremist narratives of a different focus. We chose the stimulus narrative used in the current study because emphasis—the victimization of the extremist group’s adopted constituents (i.e., civilians in HLA territory)—is a common theme in the narrative propaganda of a wide variety of groups. To be sure, several researchers have shown that perceptions of victimization increase one’s vulnerability of radicalization to violence (see Maly et al., 2013; Braddock, 2015; Kharroub, 2015; Van Den Bos, 2018; Jensen et al., 2020). Still, other kinds of narratives can have a similar effect. Future work in this domain would benefit from investigating these other types of narratives.

Moreover, we specifically created the source of the narrative stimuli (i.e., the HLA) such that no participants would feel an *a priori* affinity for the group, its cause, or its activities. Specifically, we described the source of the message to be geographically and culturally ambiguous. This decision had two key strengths. First, it controlled for any effects resulting from perceived geographic proximity or cultural affiliation with the source of the narratives before being exposed to them. Second, and most critically, it allowed us to avoid inadvertently persuading any participants about a *real* group’s ideology. These essential controls came at a cost; however, in real-world scenarios, it is likely an individual’s affinity for the source of a terrorist narrative would affect the persuasiveness of that narrative. Consider, for example, that terrorist narratives are often designed to elicit feelings of sympathy and perceptions of similarity and inclusion with the group that produces the message (see Corman and Schiefelbein, 2008; Corman, 2011). These outcomes are closely related to the persuasive efficacy of strategic messaging, meaning that without naming the author of the propaganda, there is no group to which audiences can feel sympathy, perceive similarity, or imagine inclusion.

Unfortunately, ethical concerns associated with the potential radicalization of participants precluded us from exposing them to narrative propaganda from real groups (to which they may have natural inclinations). As such, our results are limited in that they do not account for the potential effect of *a priori* affinity for a group on narrative persuasiveness. Future research in this domain would benefit from exploring this relationship, though it is likely to remain difficult to incorporate narrative propaganda from real groups into experimental research. Case analyses of specific terrorist narrative messages may be useful in this regard.

There were also psychometric considerations that may be addressed in future research. First, although the ARIS scale was conceptualized as comprising two factors (activism and radicalism), the scale was largely unidimensional in our data. This may be expected when measuring intentions with respect to terrorism, as all related activities may be perceived as non-normative and radical. However, future research may benefit from using a less overtly terroristic propaganda source to determine whether intentions derived from terrorist narrative exposure are, in fact, unidimensional. Finally, as expected, there were high correlations between the elements of the Dark Tetrad. Although this may raise alarms related to collinearity, we effectively accounted for these correlations in our predictive models.

## Data Availability Statement

The raw data supporting the conclusions of this article will be made available by the authors, without undue reservation.

## Ethics Statement

The studies involving human participants were reviewed and approved by Institutional Review Board Office of Research Protections, The Pennsylvania State University University Park, PA, United States. Written informed consent for participation was not required for this study in accordance with the national legislation and the institutional requirements.

## Author Contributions

KB contributed to the conceptualization of the study, literature review, data collection and analysis, and writeup. SS contributed to data analysis and interpretation, as well as writeup. EC and PG contributed to data interpretation and writeup. All authors contributed to the article and approved the submitted version.

## Funding

This research received funding by the VOX-Pol Center of Excellence (a European Union Framework Program 7) and The European Research Council (ERC) under the European Union’s Horizon 2020 Research and Innovation Programme (Grant 758834).

## Conflict of Interest

The authors declare that the research was conducted in the absence of any commercial or financial relationships that could be construed as a potential conflict of interest.

## Publisher’s Note

All claims expressed in this article are solely those of the authors and do not necessarily represent those of their affiliated organizations, or those of the publisher, the editors and the reviewers. Any product that may be evaluated in this article, or claim that may be made by its manufacturer, is not guaranteed or endorsed by the publisher.

## Footnotes

^1^Lee and Leets (2002) is a rare exception. The authors evaluated the explicitness of hate groups’ messages and how it affected the persuasiveness of those messages.

^2^http://www.stormfront.org.

^3^http://www.animalliberationfront.com/Saints/Authors/Stories/stories.htm.

^4^The “Homeland Liberation Alliance” does not exist; we used this name to describe the group in our stimuli for two key reasons. First, the use of a nonexistent organization avoided belief, attitude, or intention changes in relation to an actual terrorist group, an outcome we would consider unethical and unacceptable. Second, the name of the group is geographically, ethnically, and culturally non-specific. By basing the purported source of the messages from a location that could not be identified, we sought to mitigate any confounding effects of perceived geographic proximity, affinity, or affiliation.

^5^One item was removed from this subscale to bring alpha above 0.70.
