# Corrigendum: The moderating effects of “dark” personality traits and message vividness on the persuasiveness of terrorist narrative propaganda

**DOI:** 10.3389/fpsyg.2022.1013827

**Published:** 2022-11-02

**Authors:** Kurt Braddock, Sandy Schumann, Emily Corner, Paul Gill

**Affiliations:** ^1^School of Communication, American University, Washington, DC, United States; ^2^Department of Security and Crime Science, University College London, London, United Kingdom; ^3^Centre for Social Research and Methods, College of Arts and Social Sciences, Australian National University, Canberra, ACT, Australia

**Keywords:** terrorism, radicalization, narratives, narcissism, Machiavellianism, psychopathy, sadism, vividness

In the published article, there was an error. In the section *Materials and Methods, Measures, Beliefs, Attitudes, and Intentions, Behavioral Intentions*. It was reported that participants were presented with 10 items of Moskalenko and McCauley's (2009) Activism and Radicalism scale. This was not the case. Although the items used in the 10-item intention index included behaviors that overlap with behaviors mentioned in the ARIS scale, the ARIS scale itself was not used as a measurement tool for intentions in this article.

A correction has therefore been made to *Materials and Methods, Measures, Beliefs, Attitudes, and Intentions, Behavioral Intentions, Paragraph 1*. It was previously stated, “we presented them with 10 items of the Activism and Radicalism scale (ARIS; e.g., *If I lived in the HLA's territory, I would consider using deadly weapons against the HLA's enemies*; Moskalenko and McCauley, 2009).” This should be “we presented them with a 10-item index (e.g., *If I lived in the HLA's territory, I would consider using deadly weapons against the HLA's enemies*).” The corrected paragraph appears below:

Intentions represent perceived motivations to engage in specific behaviors. To gauge participants' intentions to act in support of the HLA, we presented them with a 10-item index (e.g., *If I lived in the HLA's territory, I would consider using deadly weapons against the HLA's enemies*). All items in the scale loaded on a single factor, the reliability estimate of which was good (α = 0.93).

The authors apologize for this error and state that this does not change the scientific conclusions of the article in any way. The original article has been updated.

## Publisher's note

All claims expressed in this article are solely those of the authors and do not necessarily represent those of their affiliated organizations, or those of the publisher, the editors and the reviewers. Any product that may be evaluated in this article, or claim that may be made by its manufacturer, is not guaranteed or endorsed by the publisher.
